# Clinical characteristics and factors associated with acute kidney injury among patients hospitalized with coronavirus disease: an observational retrospective study

**DOI:** 10.1590/1516-3180.2021.0668.R1.121121

**Published:** 2022-06-17

**Authors:** Edgar Dehesa-López, Adolfo Entzana Galindo, Irali María Velasco Santos, Michel Alberto Aros-Pérez, Diego Manuel Gómez Rodríguez, Erick Ojeda-Mendoza, Brenda Paola Aguilar Ide

**Affiliations:** I MD, MSc, PhD. Chief of Department of Internal Medicine, Centro de Investigación y Docencia en Ciencias de la Salud (CIDOCS), Culiacán, Mexico;; Department of Nephrology, Hospital Civil de Culiacán, Culiacán Rosales, Sinaloa, Mexico; Instituto Mexicano del Seguro Social, Culiacán, Mexico; II MD. Chief of the Hospital Epidemiological Unit, Hospital Civil de Culiacán, Culiacán, Mexico.; III MD. Internal Medicine Resident, Department of Internal Medicine, Centro de Investigación y Docencia en Ciencias de la Salud (CIDOCS), Culiacán, Mexico.; IV MD. Internal Medicine Resident, Department of Internal Medicine, Centro de Investigación y Docencia en Ciencias de la Salud (CIDOCS), Culiacán, Mexico.; V MD. Internal Medicine Resident, Department of Internal Medicine, Centro de Investigación y Docencia en Ciencias de la Salud (CIDOCS), Culiacán, Mexico.; VI MD. Internal Medicine Resident, Department of Internal Medicine, Centro de Investigación y Docencia en Ciencias de la Salud (CIDOCS), Culiacán, Mexico.; VII MD. House Staff, Department of Internal Medicine, Centro de Investigación y Docencia en Ciencias de la Salud (CIDOCS), Culiacán, Mexico.

**Keywords:** Acute kidney injury, COVID-19, Community-acquired infections, Mortality, Risk factors, Acute renal injuries, Coronavirus disease 19, Hospital-acquired AKI, Community-acquired AKI

## Abstract

**BACKGROUND:**

Coronavirus disease 19 (COVID-19) is a multisystemic disease with high incidence of acute kidney injury (AKI).

**OBJECTIVE:**

To describe the clinical characteristics and factors associated with AKI among patients hospitalized with COVID-19.

**DESIGN AND SETTING:**

Retrospective cohort conducted at Hospital Civil de Culiacan, Mexico.

**METHODS:**

We included 307 patients hospitalized due to COVID-19. AKI was defined and staged based on serum creatinine levels in accordance with the criteria of the Acute Kidney Injury Network (AKIN). Multivariate logistic regression analysis was used to determine factors associated with AKI.

**RESULTS:**

The patients’ age was 56 ± 15 years (64.5% male). The incidence of AKI was 33.6% (n = 103). Overall, 53.4% of patients had community-acquired AKI, and 46.6% had hospital-acquired AKI. Additionally, 15.5% of them presented AKIN stage 1; 34% had AKIN stage 2; and 50.5% had AKIN stage 3. Hemodialysis was required for 10.7% of the patients. The factors associated with AKI were chronic kidney disease (odds ratio, OR: 10.8; P = 0.04), use of norepinephrine (OR: 7.3; P = 0.002), diabetes mellitus (OR: 2.9; P = 0.03), C-reactive protein level (OR: 1.005; P = 0.01) and COVID-19 severity index based on chest tomography (OR: 1.09; statistical trend, P = 0.07). Hospital stay (11 ± 7 days; P < 0.001) and mortality (83.5 versus 31.4%; P < 0.05) were greater among patients with AKI.

**CONCLUSION:**

AKI was a frequent and serious complication in our cohort of patients hospitalized with COVID-19, which was associated with high mortality and long hospital stay.

## INTRODUCTION

Coronavirus disease 19 (COVID-19) was initially considered to predominantly be a pulmonary disease. However, it is now known that it is actually a disease with a wide spectrum of clinical manifestations and frequent multisystem involvement, especially in severe cases.^1^ From a pathophysiological viewpoint, entry of severe acute respiratory syndrome coronavirus-2 (SARS-CoV-2) into cells occurs through the receptor for the angiotensin-converting enzyme 2, which is highly expressed in podocytes and the apical border of epithelial cells of the kidney proximal tubules. This could explain the kidney trophism exhibited by SARS-CoV-2.^2^

The reported incidence of acute kidney injury (AKI) among patients with COVID-19 has been variable, depending on the diagnostic criteria used, geographical region and clinical context. In patients hospitalized due to COVID-19, the reported incidences of AKI range from 0% to 57% in different published studies.^3^ Clinical and pathological studies on patients with COVID-19 who developed AKI have documented various clinical and histological findings, such as hematuria, proteinuria, focal and segmental glomerulosclerosis, tubular pigment deposition, tubulointerstitial nephritis, fibrosis and tubular atrophy, evidence of hypoperfusion, thrombotic microangiopathy and acute tubular necrosis.^4^ ^,^ ^5^ ^,^ ^6^ On the other hand, the negative effect of AKI on hospitalized patients with COVID-19 has been consistently documented and has been found to be associated with a long hospital stay, mechanical ventilation requirement, high medical care costs and mortality.^3^ ^,^ ^7^ ^,^ ^8^ The factors associated with the development of AKI among these patients, previously reported in the literature, are male sex, previous diagnosis of chronic kidney disease (CKD), diabetes, hypertension, use of vasopressors, use of mechanical ventilation and presence of markers for disease activity (ferritin, D-dimer and C-reactive protein [CRP]) and severity (partial pressure of oxygen [PaO2]/fraction of inspired oxygen [FiO2] and Sequential Organ Failure Assessment [SOFA] score).^3^ ^,^ ^7^ ^,^ ^9^

## OBJECTIVE

Because of the limited information published on this topic in Mexico, the main objective of our study was to describe the clinical presentation, associated factors and prognosis of AKI among patients hospitalized for COVID-19 in this country.

## METHODS

### Study design and population

This was a single-center, retrospective and observational cohort study. The study protocol was reviewed and approved (registration number: 1/386/193; date: August 31, 2021) by the institutional ethics committee of our hospital; and it fulfilled the international ethical standards of the Declaration of Helsinki. The need for written informed consent was waived because of the observational nature of the study.

**Figure 1.**
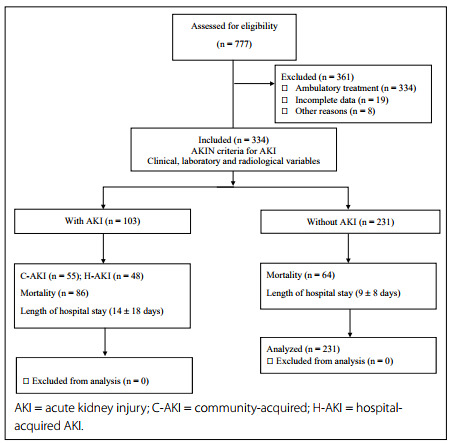
Patient inclusion flowchart.

We included 307 patients hospitalized due to severe COVID-19 in the internal medicine service between March 18, 2020, and September 11, 2020. Patients older than 18 years of age, of both sexes, and patients with a diagnosis of severe COVID-19 were included. Pregnant women, patients with end-stage CKD, patients on chronic dialysis (hemodialysis or peritoneal dialysis) prior to admission, patients hospitalized for less than 24 hours, cases of mild/moderate COVID-19 and those with incomplete collection of data on the variables studied were excluded (Figure 1).

### Data collection and definitions

Clinical variables (age, sex, comorbidities, oxygen saturation and type of respiratory support), radiological variables (severity index based on chest computed tomography [CT]) and laboratory variables (glucose, urea, creatinine, hematic biometry, sodium, potassium, arterial blood gas, CRP, ferritin serum, D-dimer and procalcitonin) were collected at admission and during hospitalization every 24 to 48 hours. The definitive diagnosis of COVID-19 was integrated based on the polymerase chain reaction test results, chest CT findings and serum levels of CRP, ferritin, D-dimer and procalcitonin. AKI was diagnosed and staged at admission (community-acquired AKI [C-AKI]) or during hospitalization (hospital-acquired AKI [H-AKI]) based on the serum creatinine level, in accordance with the criteria of the Acute Kidney Injury Network (AKIN).^10^ The type of AKI (C-AKI/H-AKI), dialysis requirement and evolution (transitory AKI, persistent AKI or acute kidney disease [AKD]) of each episode of AKI were studied in accordance with the criteria of the Acute Disease Quality Initiative.^11^ Transitory AKI was defined as a complete reversal of AKI in accordance with Kidney Disease Improving Global Outcomes (KDIGO) criteria within 48 hours of AKI onset. Persistent AKI was defined as continuance of AKI, using serum creatinine criteria, beyond 48 hours after AKI onset. AKD was defined as a condition in which AKI stage 1 or greater was present for ≥ 7 days after an AKI-initiating event. Kidney recovery was defined as a return to baseline creatinine. CKD was defined in accordance with the KDIGO CKD guidelines, based on previously documented findings or during the evaluation for COVID-19. Oliguria was defined as urinary output < 400 ml/day at AKI diagnosis. The impact of AKI on prognosis was studied by comparing mortality and hospital stay (in days) between patients with and without AKI.

### Statistical analysis

Descriptive statistics with means/standard deviations or median/interquartile range were used to describe continuous variables according to distribution data; and frequencies and proportions were used to describe categorical variables. Comparisons between pairs of groups were performed using Student’s t test or the Mann-Whitney U test for continuous variables according to distribution data; and the χ^2^ test was used for categorical variables. Comparisons between more than two groups were performed using the Kruskal-Wallis test.

The clinical impact of AKI was evaluated based on in-hospital mortality and the length of hospital stay. Factors associated with the development of AKI were analyzed using multivariate logistic regression analysis. All clinically relevant variables with P-values < 0.05 in bivariate analysis were included for entry into multivariate modeling. Differences were considered statistically significant at P < 0.05.

Data analysis was performed using the IBM SPSS Statistics software for Macintosh, version 22.0 (IBM Corp., Armonk, New York, United States). No formal sample size calculation was carried out, because of the observational and convenience-sampling nature of the study.

## RESULTS

### General characteristics of the population studied

During the study period, 777 patients were treated for COVID-19 in our hospital, of whom 443 were treated on an outpatient basis and 334 required hospitalization in the internal medicine service. Among the latter patients, who were assessed for eligibility, three patients were excluded because they were younger than 18 years of age and another 24 adult patients were excluded (five who were hospitalized for less than 24 hours and 19 whose data were incomplete). Therefore, 307 patients were included in the final analysis of our study (Figure 1).

The patients’ age was 56 ± 16 years, and males were more affected (64.5%, n = 198). The most frequently observed comorbidities in our population were hypertension (41.4%; n = 127), diabetes mellitus (30.9%; n = 95) and smoking (16.9%; n = 52). The remaining characteristics are shown in Table 1.

**Table 1.** General population characteristics

Variables	n = 307	%
Age (years)	56 ± 15	
Gender (female/male)	109 (35.5%) / 198 (64.5%)	
Diabetes mellitus	95	30.9%
Hypertension	127	41.4%
Chronic pulmonary obstructive disease	6	2.0%
Chronic kidney disease	15	4.9%
Heart failure	16	5.2%
Smoking	52	16.9%
**Respiratory support**		
Simple mask	54	17.6%
Reservoir mask	109	35.4%
High-flow nasal cannula	10	3.3%
Non-invasive ventilation	26	8.5%
Invasive ventilation	108	35.2%

### Clinical characteristics and evolution of AKI episodes

The frequency of AKI in our population was 33.6% (n = 103), of which 53.4% (n = 55) corresponded to C-AKI and 46.6% (n = 48) corresponded to H-AKI. The most frequently observed stage of severity was AKIN stage 3 in 50.5% (n = 52) of the cases, followed by AKIN stage 2 (34%; n = 35) and AKIN stage 1 (15.5%; n = 16). Among the AKI episodes, 48.5% (n = 50) were oliguric, and 10.7% (n = 11) required renal replacement therapy, which consisted of intermittent hemodialysis in all cases.

At the time of hospital discharge or death, only 35.9% (n = 37) of the patients with AKI had achieved recovery of kidney function. On the other hand, 25.2% (n = 26) of the patients had transitory AKI, 36.9% (n = 38) had persistent AKI and 37.9% (n = 39) had AKD.

In comparing the characteristics according to the type of AKI (transitory AKI, persistent AKI and AKD), we observed that the frequency of oliguria was higher among patients with AKD than among those with either transitory AKI or persistent AKI (65.6% versus 29.6% versus 13.3%, respectively; P < 0.001). The frequency of AKIN 3 episodes was also higher among patients with AKD than among those with either persistent AKI or transitory AKI (72.1% versus 33.3% versus 14.8%, respectively; P < 0.001). In contrast, the frequency of AKIN 1 episodes was higher in patients with transitory AKI than among those with either persistent AKI or AKD (44.4% versus 20% versus 3.3%, respectively; P < 0.001).

Recovery of kidney function was more frequent among patients with transitory AKI than among those with either persistent AKI or AKD (85.2% versus 60% versus 4.9%, respectively; P < 0.001). There were no statistically significant differences in the frequency of hemodialysis between patients with transitory AKI, persistent AKI or AKD (7.4% versus 0% versus 14.8%, respectively; P = 0.253).

### Comparison of clinical and laboratory characteristics between patients with and without AKI

In comparing the general characteristics between the groups, we observed that patients with AKI were older than those without AKI (61 ± 14 versus 54 ± 15 years; P = 0.001) and had higher frequencies of diabetes mellitus (44.7% versus 24%; P < 0.05), hypertension (58.3% versus 32.8%; P < 0.05), CKD (13.6% versus 0.5%; P < 0.05) and heart failure (9.7% versus 2.9%; P < 0.05). On the other hand, the frequencies of use of invasive mechanical ventilation (65% versus 20.1%; P < 0.05) and use of norepinephrine (56.3% versus 10.8%; P < 0.05) were also higher among patients with AKI than among those without.

Patients with AKI had higher total leukocyte counts (13,710 versus 10,330 /μl; P < 0.001), serum glucose levels (146 versus 125 mg/dl; P = 0.001), urea levels (57 versus 34 mg/dl; P = 0.001), serum creatinine levels (1.3 versus 0.7 mg/dl; P < 0.001), serum potassium levels (4.5 versus 4.2 mEq/l/; P = 0.019), serum CRP levels (109 versus 95 mg/dl; P = 0.025), ferritin levels (848 versus 615 ­ng/­dl; P < 0.001), D-dimer levels (1,420 versus 760 ­ng/­dl; P < 0.001) and severity score based on chest CT (24 versus 19; P < 0.001) at admission, than patients without AKI. In addition, patients with AKI presented lower pH (7.42 versus 7.46; P < 0.001), and PaO2/FiO2 index (124 versus 170; P = 0.005) than those without AKI. The remaining characteristics are shown in Tables 2 and 3.

**Table 2.** Comparison of general characteristics between patients with and without acute kidney injury (AKI)

Variables	Without AKI		With AKI		P
	n = 204	%	n = 103	%	
Age (years)	54 ± 15		61 ± 14		< 0.001
Gender (female/male)	74 (36.3%) / 130 (63.7%)		35 (34%) / 68 (66%)		0.707
Diabetes mellitus	49	24.0%	46	44.7%	< 0.001
Hypertension	67	32.8%	60	58.3%	< 0.001
CPOD	5	2.5%	1	1.0%	0.668
CKD	1	0.5%	14	13.6%	< 0.001
Heart failure	6	2.9%	10	9.7%	0.026
Smoking	31	15.2%	21	20.4%	0.263
**Respiratory support**					
Simple mask	49	24.0%	5	4.9%	< 0.001
Reservoir mask	92	45.1%	17	16.5%	
HFNC	6	2.9%	4	3.9%	
NIV	16	7.8%	10	9.7%	
IV	41	20.1%	67	65.0%	

CPOD = chronic pulmonary obstructive disease; CKD = chronic kidney disease; HFNC = high-flow nasal canula; NIV = noninvasive ventilation; IV = invasive ventilation.

**Table 3.** Comparison of clinical and laboratory characteristics at admission between patients with and without acute kidney injury (AKI)

Variables	Without AKI		With AKI		P
	Median	IQR / %	Median	IQR / %	
Hemoglobin (g/dl) (X/SD)	13.7	(12.6-14.8)	13.5	(11.5-14.5)	0.689
White blood cells (/μl) (x1000)	10.3	(7.7-14.8)	13.7	(9.8-19.7)	< 0.001
Total lymphocytes (/μl)	950	(578-1,322)	924	(568-1,551)	0.571
Platelets (/μl) (x1000)	244	(187-307)	263	(210-332)	0.333
Serum glucose (mg/dl)	125	(96-169)	146	(120-210)	0.001
Serum creatinine (mg/dl)	0.7	(0.6-0.8)	1.3	(0.8-2.1)	< 0.001
C-reactive protein (mg/dl)	95	(48-185)	109	(83-192)	0.025
Ferritin (ng/dl)	615	(349-904)	848	(471-1,000)	< 0.001
D-dimer (ng/dl)	760	(365-1,965)	1,420	(670-4,140)	< 0.001
Procalcitonin (ng/dl)	0.10	(0.05-0.26)	0.29	(0.11-1.09)	< 0.001
PaO2/FiO2	170	(101-266)	124	(84-180)	0.005
Index CT severity	19	(14-22)	24	(21-25)	< 0.001
Invasive ventilation	41	20.1%	67	65.0%	< 0.001
Use of norepinephrine	22	10.8%	58	56.3%	< 0.001

IQR = interquartile range; SD = standard deviation; CT = chest tomography; PaO2 = partial pressure of oxygen; FiO2 = fraction of inspired oxygen.

### Comparison of clinical and laboratory characteristics between patients with C-AKI and those with H-AKI

The frequency of AKI was 33.6% (n = 103), of which 53.4% (n = 55) corresponded to C-AKI and 46.6% (n = 48) to H-AKI. Compared with patients with C-AKI, patients with H-AKI required mechanical ventilation more frequently (81.3% versus 50.9%; P = 0.018). On the other hand, patients with C-AKI had higher total leukocyte counts (13,950 versus 10,940 /μl; P = 0.037), serum glucose levels (164 versus 127 mg/dl; P = 0.001), ferritin levels (854 versus 648 ng/dl; P = 0.009), D dimer levels (1.627 versus 847 ng/dl; P = 0.020), procalcitonin levels (0.5 versus 0.1 ng/dl; P = 0.001) and severity score based on chest CT (24 versus 20; P = 0.003) at admission, than patients with H-AKI.

We did not observe any statistically significant differences in relation to age, comorbidities, disease severity markers, severity of AKI episodes, dialysis requirement or recovery of kidney function, between the C-AKI and H-AKI groups (Table 4).

**Table 4.** Comparison of clinical and laboratory characteristics at admission between patients with community-acquired acute kidney injury (C-AKI) and those with hospital-acquired acute kidney injury (H-AKI)

Variables	C-AKI (n = 55)		H-AKI (n = 48)		P
	n/median	%/IQR	n/median	%/IQR	
General characteristics:					
Age (years) (mean ± SD)	61 ± 15		60 ± 14		0.678
Female	18	32.7%	17	35.4%	0.77
Male	37	67.3%	31	64.6%	
Diabetes mellitus	28	50.9%	18	37.5%	0.17
Hypertension	36	65.5%	24	50.0%	0.11
Chronic kidney disease	7	12.7%	7	14.6%	0.78
Respiratory support:					
Simple mask	4	7.3%	1	2.1%	0.018
Reservoir mask	14	25.5%	3	6.3%	
High-flow nasal canula	2	3.6%	2	4.2%	
NIV	7	12.7%	3	6.3%	
IV	28	50.9%	39	81.3%	
AKI characteristics:					
AKIN 1	10	18.2%	6	12.5%	0.32
AKIN 2	21	38.2%	14	29.2%	
AKIN 3	24	43.6%	28	58.3%	
Hemodialysis	7	12.7%	4	8.3%	0.47
Recovered kidney function	19	34.5%	18	37.5%	0.75
Laboratory characteristics:					
Hemoglobin (g/dl)	12.5	(10.4-14.1)	13.8	(12.5-14.8)	0.027
Total leucocytes (/μl) (x1000)	13.9	(9.8-20.9)	10.9	(7.9-15.6)	0.037
Total lymphocytes (/μl)	857	(488-1,256)	961	(595-1,382)	0.571
Platelets (/μl) (x1000)	250	(221-318)	253	(188-311)	0.999
Serum creatinine (mg/dl)^*^	1.8	(1.3-2.8)	1.4	(1.3-2.1)	0.256
C-reactive protein (mg/dl)	120	(48-192)	96	(48-192)	0.173
Ferritin (ng/dl)	854	(453-1,000)	648	(361-964)	0.009
D-dimer (ng/dl)	1627	(755-3,970)	847	(390-2,090)	0.020
PaO2/FiO2 at admission	140	(102-244)	142	(100-236)	0.875
Index CT severity	24	(21-25)	20	(15-24)	0.003
Use of norepinephrine	28	50.9	30	62.5	0.320

IQR = interquartile range; SD = standard deviation; NIV = noninvasive ventilation; IV = invasive ventilation; AKIN = acute kidney injury network. PaO2 = partial pressure of oxygen; FiO2 = fraction of inspired oxygen. ^*^Serum creatinine at the time of AKI diagnosis.

### Factors associated with development of AKI among patients hospitalized due to COVID

In our study, the factors independently associated with AKI were as follows: previous diagnosis of CKD (odds ratio, OR: 10.8; 95% confidence interval, CI: 1.02-116.1; P = 0.04), use of norepinephrine (OR: 7.3; 95% CI: 2.1-25.8; P = 0.002), diabetes mellitus (OR: 2.9; 95% CI: 1.05-8.3; P = 0.03), serum CRP level (OR: 1.005; 95% CI: 1.001-1.009; P = 0.01) and COVID severity index based on chest CT (OR: 1.09; 95% CI: 0.99-1.21; statistical trend, P = 0.07) (Table 5).

**Table 5.** Multivariate analysis on factors associated with acute kidney injury among patients hospitalized due to coronavirus disease

Variables	OR	95% CI		P
		Lower	Upper	
Age (years)	1.030	0.991	1.071	0.133
Gender (male/female)	1.692	0.619	4.627	0.305
Diabetes mellitus (yes/no)	2.978	1.058	8.385	0.039
Hypertension (yes/no)	0.943	0.322	2.762	0.914
Chronic kidney disease (yes/no)	10.892	1.022	116.112	0.048
Invasive ventilation (yes/no)	1.496	0.416	5.375	0.537
Use of norepinephrine (yes/no)	7.369	2.104	25.816	0.002
C-reactive protein (mg/dl)	1.005	1.001	1.009	0.010
Index CT severity (points)	1.099	0.991	1.218	0.073

OR = odd ratio; CT = chest tomography; CI = confidence interval.

### Clinical impact of AKI on the prognosis of patients hospitalized due to COVID-19

Overall mortality was 48.9% (n = 150), and this was higher among patients with AKI than in those without AKI (83.5% versus 31.4%; P = 0.001). No statistically significant difference in mortality was observed between patients with C-AKI and those with H-AKI (80% and 87.5%, respectively; P = 0.42). On the other hand, the median length of hospitalization in our population was 8 days (range: 3-15 days), which was longer among patients with AKI than among those without AKI (11 versus 7 days; P < 0.001), but not between patients with H-AKI and those with C-AKI) (8 versus 8 days; P = 0.918).

In comparing mortality according to the type of AKI (transitory AKI, persistent AKI and AKD), we observed that, among these three types, mortality was only higher among patients with AKD (74.1% versus 66.7% versus 91.8%, respectively; P < 0.015). No statistically significant difference in the length of hospitalization was observed between patients with transitory AKI, persistent AKI and AKD (15 versus 10 versus 10 days, respectively; P = 0.285).

## DISCUSSION

COVID-19 is considered to be a lung disease; however, its clinical and systemic spectrum is very broad, from asymptomatic cases to severe cases with multisystemic disease, including kidney damage. As in the rest of the world, in our retrospective cohort of patients hospitalized due to COVID-19, AKI was a frequent complication, observed in 33.6% of the cases.

The incidence of AKI among patients hospitalized due to COVID-19 reported by other authors has varied according to the diagnostic criteria for AKI, geographical region and clinical context studied. In a meta-analysis by Lin et al. on 79 studies that included 49,692 patients with COVID-19 from Asia, Europe and North America, the incidences of AKI were 22.6% in North America, 11.6% in Europe and 4.3% in Asia.^3^ In contrast, Chen et al. conducted a systematic review of 20 studies with 6,495 patients hospitalized due to COVID-19 in China, Italy, the United Kingdom and the United States. The reported incidence of AKI was 8.9% (95% CI: 4.6-14.5%) with a range from 0 to 57.1% in the different studies included.^12^ In another meta-analysis on 40 studies with 24,377 patients hospitalized due to COVID-19, Shao et al. reported that the incidence of AKI was 10% (95% CI: 8-13%) with a range from 0.5 to 49.3%.^8^ Lastly, Martinez-Rueda et al. reported that the incidence of AKI was 30% in a cohort of 1,170 Mexican patients hospitalized due to COVID-19.^9^

Another important finding from our study was that AKIN stage 3 of AKI occurred most frequently, followed by stage 2 and stage 1, which indicates that AKI was a frequent and serious complication in our population. Regarding the severity of AKI episodes, the data published by other authors have varied according to the region and the clinical context studied. In a study by Chan et al. on 3,993 patients hospitalized due to COVID-19 in five hospitals in New York, the incidences of AKI were 46% in the general hospitalized population and 76% in patients in the intensive care unit. AKIN stage 3 occurred most frequently overall (AKIN stage 3 = 42%; AKIN stage 2 = 19%; and AKIN stage 1 = 39%) and among intensive care patients (AKIN stage 3 = 56%; AKIN stage 2 = 17%; and AKIN stage 1 = 28%).^13^ However, in one of the largest cohorts of patients hospitalized due to COVID-19 (n = 5,449), the incidence of AKI was 36.6%. AKIN stage 1 occurred most frequently (46.5%), followed by AKIN stage 2 (22.4%) and AKIN stage 3 (31.1%).^7^

Recently, there has been great interest in differentiating between C-AKI and H-AKI among patients hospitalized due to COVID-19, given the different etiology and prognosis between these two types of AKI.^9^ ^,^ ^14^ In our cohort, patients with H-AKI had respiratory failure more frequently and patients with C-AKI had higher levels of activity markers (D-dimer and ferritin) and severity markers (index CT severity) for the disease. We did not observe any statistically significant difference in relation to age, comorbidities, severity of AKI episodes, dialysis requirement, recovery of kidney function or mortality, between patients with C-AKI and those with H-AKI. Our findings contrast with those reported by Martinez-Rueda et al. in their cohort of Mexican patients hospitalized due to COVID-19. Although C-AKI (64.1%) was also the more frequent type in their study and they did not observe any differences in mortality between patients with C-AKI and those with H-AKI (53% and 50%, respectively; P = 0.65), the patients with C-AKI were older and had greater levels of comorbidities (based on the Charlson index) than the patients with H-AKI, who were younger, had greater multiorgan failure (higher SOFA score), greater respiratory failure (lower PaO2/FiO2 index), greater severity of AKI episodes (AKIN stages 2-3), and greater dialysis requirement (27% versus 7%; P = 0.001).^9^

In our population, the overall frequency of dialysis required was 10.7%, with no statistically significant difference between the C-AKI and H-AKI groups. The frequency reported by other researchers has varied widely worldwide, from 0.4% to 22.3%.^3^ ^,^ ^8^ ^,^ ^15^ This variability in dialysis requirement worldwide could partly be explained by differences in the severity of AKI episodes and the clinical context studied, as well as in the availability and prioritization of dialysis treatment assignments during the pandemic, due to oversaturation of medical services and the poor prognosis of patients with COVID-19 and AKI, which may have underestimated the true frequency of the dialysis requirement among these patients.

In our study, the history of CKD, use of norepinephrine, presence of diabetes mellitus, serum CRP level and COVID severity index based on chest CT were the factors associated with development of AKI. In this regard, multiple factors have been associated with development of AKI, as referenced by other authors. In a retrospective cohort on 5,449 patients hospitalized due to COVID-19 in New York, Hirsch et al. reported that age, black race, presence of diabetes mellitus, arterial hypertension, cardiovascular disease, mechanical ventilation use and vasopressor use were factors associated with development of AKI.^7^ Hamilton et al., in a retrospective cohort of 1,032 patients hospitalized due to COVID-19 in the United Kingdom, reported that male sex, presence of CKD, presence of diabetes mellitus and serum CRP level were factors associated with AKI.^16^ In a meta-analysis on 26 studies with 5,497 patients hospitalized due to COVID-19, Hansrivijit et al. reported that age, hypertension, presence of diabetes mellitus and serum creatinine level were risk factors associated with AKI.^15^ Lastly, Martínez-Rueda et al., in a prospective cohort of 1,170 Mexican patients hospitalized due to COVID-19, reported that certain factors were specific for the type of AKI. The Charlson index, CKD, SOFA score, serum glucose level, creatinine level, CRP level and troponin level were factors associated with C-AKI, while the body mass index, glucose level, troponin level and intubation were factors associated with development of H-AKI.^9^

The overall fatality rate in our population was 48.9%, and it was higher among patients with AKI than among those without AKI. In addition, the median hospital stay was longer among patients with AKI than among those without AKI. It has been consistently demonstrated that AKI has a negative impact on the prognosis of patients hospitalized due to COVID-19 and is associated with a long hospital stay, high mechanical ventilation requirements and fatality. In a meta-analysis on 40 studies with 24,527 patients, Shao et al. reported that the overall fatality rate was 20.3%, and that it was higher among patients with AKI than among those without AKI (63.1% versus 12.9%; P < 0.01), with an OR for mortality of 14.6 (95% CI: 9.94-21.5; P < 0.00001).^8^ On the other hand, similar to the findings of Martínez-Rueda et al.,^9^ we did not observe any statistically significant difference in mortality between patients with C-AKI and those with H-AKI. However, our fatality rate was above the overall fatality rate (48.9% versus 27%), C-AKI fatality rate (80% versus 53%) and H-AKI fatality rate (87.5% versus. 50%) reported by Martínez-Rueda et al.^9^ The great variability in the prognosis of these patients observed worldwide might be partially explained by differences in the severity of the patients’ conditions, the clinical contexts studied, the hospital resources and infrastructure and the availability of trained and specialized personnel for caring for these patients, among different hospitals during the pandemic.

The present study had some limitations. Firstly, it was a retrospective study conducted in a single center. Therefore, it was not possible to include the criterion of urinary volume for diagnosing AKI, which could have underestimated the frequency of AKI in our population. Moreover, the precise cause of each AKI episode could not be determined. Secondly, we did not include variables relating to treatment in our analysis because of the great variability and modifications of the drugs used during the pandemic. Lastly, it was not possible to construct a specific logistic regression model of factors associated with C-AKI and H-AKI because of the small number of cases. Nevertheless, despite these deficiencies, we believe that the results from our study are valid and useful for improving the characterization of AKI episodes among patients hospitalized due to COVID-19.

## CONCLUSION

AKI was a frequent and serious complication in our cohort of patients hospitalized due to COVID-19, which was associated with high mortality and long hospital stay. This highlights the importance of close nephrological surveillance for early detection of patients at high risk of developing AKI.
